# Highly Accurate Structure-Based Prediction of HIV-1 Coreceptor Usage Suggests Intermolecular Interactions Driving Tropism

**DOI:** 10.1371/journal.pone.0148974

**Published:** 2016-02-09

**Authors:** Chris A. Kieslich, Phanourios Tamamis, Yannis A. Guzman, Melis Onel, Christodoulos A. Floudas

**Affiliations:** 1 Artie McFerrin Department of Chemical Engineering, Texas A&M University, College Station, TX, United States of America; 2 Texas A&M Energy Institute, Texas A&M University, College Station, TX, United States of America; 3 Department of Chemical and Biological Engineering, Princeton University, Princeton, NJ, United States of America; University of British Columbia, CANADA

## Abstract

HIV-1 entry into host cells is mediated by interactions between the V3-loop of viral glycoprotein gp120 and chemokine receptor CCR5 or CXCR4, collectively known as HIV-1 coreceptors. Accurate genotypic prediction of coreceptor usage is of significant clinical interest and determination of the factors driving tropism has been the focus of extensive study. We have developed a method based on nonlinear support vector machines to elucidate the interacting residue pairs driving coreceptor usage and provide highly accurate coreceptor usage predictions. Our models utilize centroid-centroid interaction energies from computationally derived structures of the V3-loop:coreceptor complexes as primary features, while additional features based on established rules regarding V3-loop sequences are also investigated. We tested our method on 2455 V3-loop sequences of various lengths and subtypes, and produce a median area under the receiver operator curve of 0.977 based on 500 runs of 10-fold cross validation. Our study is the first to elucidate a small set of specific interacting residue pairs between the V3-loop and coreceptors capable of predicting coreceptor usage with high accuracy across major HIV-1 subtypes. The developed method has been implemented as a web tool named CRUSH, CoReceptor USage prediction for HIV-1, which is available at http://ares.tamu.edu/CRUSH/.

## Introduction

In recent years, significant advances in the treatment of human immunodeficiency virus type 1 (HIV-1) have been made, and one class of drugs that has contributed to that success is inhibitors that target chemokine receptors CCR5 and CXCR4, collectively known as the HIV-1 coreceptors [1]. For some HIV-1 viral strains these therapeutics, including maraviroc, are able to circumvent the difficulties of thwarting the quickly mutating HIV-1 by targeting host cell coreceptors and inhibiting a key interaction with the third variable region of HIV-1 gp120 (V3-loop) necessary for entry into host cells. The situation is further complicated by HIV-1 tropism, or the ability of the virus to change the cell type infected, with the transition from a CCR5-specific (R5) virus to a CXCR4-specific (X4) virus often indicating a progression to advanced stages of infection for subtype B viruses [2]. Therefore, tropism determination is performed in conjunction with coreceptor inhibitors to ensure the success of a treatment regimen. Phenotypic methods, such as the Trofile assay, can be costly with a slow turn-around. As an alternative, genotypic methods based on sequencing the V3-loop and using bioinformatics methods to predict coreceptor usage can also be used [3,4].

Ever since the HIV-1 coreceptors were identified [5–8], there has been significant interest in understanding what drives HIV-1 coreceptor usage. Multiple rules have been established to predict the transition from an R5- to an X4-virus. Increase in the positive net charge of the V3-loop has been shown to favor CXCR4 (referred as Rule I) [9–11], as does a positively charged residue at V3-loop positions 11, 24, or 25 (so-called 11/24/25 rule, referred here as Rule II) [12]. Additionally, the loss of a highly conserved glycosylation motif (referred here as Rule III) found at V3-loop positions 6–9 is also associated with an X4-virus [13]. A simple statistical model has been proposed that combines the three established rules to provide probabilities for HIV-1 coreceptor usage given binary/discrete values for the rules [9]. More elaborate bioinformatics methods have also been previously developed that provide improved accuracy and sensitivity [14–18]. For more details regarding bioinformatics analysis of HIV-1, the authors refer to the review by Aiamkitsumrit et al. [19].

With the availability of x-ray crystallographic structures of the V3-loop [20,21], a new generation of structure-based methods have been developed [22–24]. Sander et al. [22] introduced structural descriptors that described the spatial arrangement of functional groups within V3-loop sequences based on binned distance distributions. A more elaborate method based on a discretized description of the electrostatic hull surrounding the V3-loop was developed by Dybowski et al. [23]. Most recently, Bozek et al. [24] utilized an approach similar to that used by Sander et al., but instead utilized the values of 54 amino acid indices mapped to spheres representing each V3-loop sequence. However, none of these methods, nor to the best of our knowledge any other existing methods, utilize structural details of the specific interactions between the V3-loop and chemokine receptors CCR5/CXCR4 to predict HIV-1 coreceptor usage.

**Fig 1:**
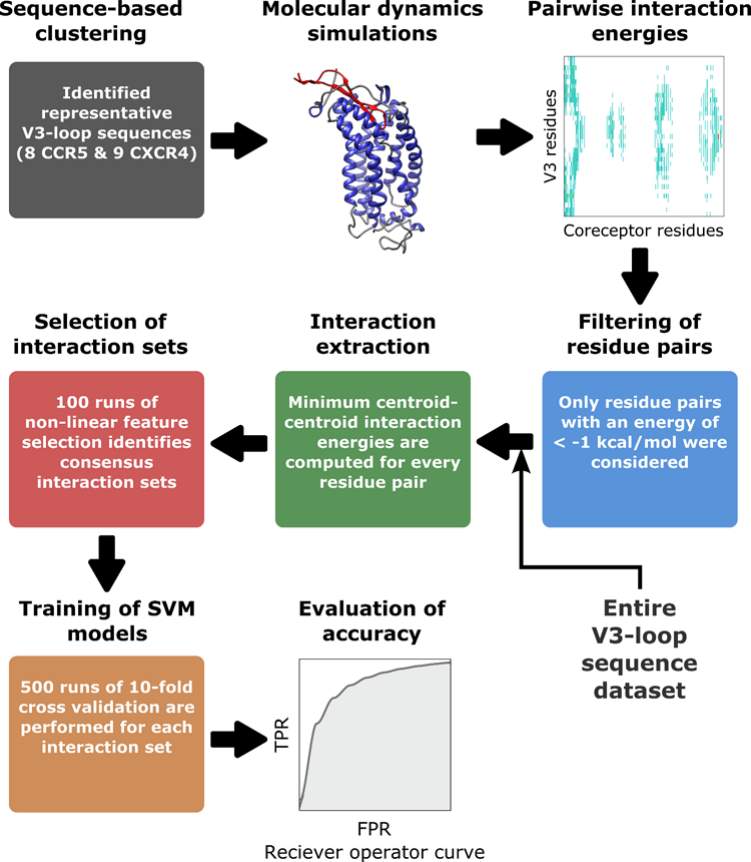
Flowchart of hybrid modeling approach.

Recently, structural data regarding the interactions between the HIV-1 proteins and their ligands [25–28], including computationally derived structures of CCR5/CXCR4:V3-loop complexes developed by our group [29,30], have provided molecular level details of HIV-1. However, to date, the specific interactions driving tropism have yet to be identified. Even in the context of the recent insights into the structure of V3-loop:coreceptor complexes [29,30], prediction of HIV coreceptor usage remains a highly complex problem. Capturing the effects of V3-loop mutations on coreceptor usage first requires a selection of key V3-loop:coreceptor interactions out of the thousands possible, since interactions driving coreceptor usage may be energetically small and long-range. Efficient modeling of the energetics of the interactions is also important, in order to allow the analysis of the thousands of known V3-loop sequences. Furthermore, GPCRs are highly structurally flexible, which implies that different V3-loop sequences could have different binding modes. To this end, we have developed a multifaceted hybrid approach for investigating the interactions that drive coreceptor usage, using tools from computational biophysics, structural bioinformatics, and machine learning, as illustrated by Fig 1. Our proposed method is of similar spirit to other recent computational methods that combine potential energy calculations with machine learning to investigate protein interactions [31,32]. Molecular dynamics (MD) simulations were used to investigate structural and physicochemical variability of V3-loop:coreceptor complexes. Biophysical insights were converted into structural bioinformatics features using a statistical centroid-centroid force field [33]. Nonlinear support vector machines (SVM) were trained to predict coreceptor usage based on the four rules (Net charge, 11/24/25, motif, length), and extracted V3-loop:coreceptor interactions. Finally, a novel non-linear feature selection algorithm was used to narrow down the necessary and sufficient V3-loop:coreceptor interacting pairs.

## Methods

### Dataset

A combination of V3-loop sequences from the Los Alamos HIV database (downloaded on April 17, 2014) and datasets published in three previous studies was used (Dybowski et al. [23], Bozek et al. [24], and Sander et al. [22]) was collected, which contained 824 CXCR4 tropic and 8389 CCR5 tropic sequences in total. The data was filtered by first identifying unique sequences only, and by removing sequences with special characters. V3-loop sequences that did not begin and end in cysteine were also removed. Finally, sequences with contradicting coreceptor usage in different sets/patients were also removed. This resulted in a superset of non-redundant V3-loop sequences containing 235 CXCR4 tropic and 2220 CCR5 tropic sequences that were used for all training and testing of SVM models, which is provided in S1 Dataset. Since phylogenetic relationships between sequences derived from the same patient may bias observed accuracies, we have also identified a subset of our superset, which contains one sequence per patient and is referred to as the unique patient subset. The unique patient subset contains 114 CXCR4 tropic and 967 CCR5 tropic sequences, and is provided in S2 Dataset.

### Molecular dynamics and interaction energies

The starting points for our molecular modeling were the computationally-derived structures of CCR5/CXCR4:V3-loop complexes that were previously developed by our group using a framework that combines rigid protein docking and molecular dynamics simulations [29,30,34]. CD-HIT Suite [35] was used to select clusters of representative HIV-1 gp120 V3 loop sequences based on the Los Alamos dataset. Eight CCR5 recognizing and nine CXCR4 recognizing HIV-1 gp120 V3 loop sequences were selected. We initially modeled the structures of the representative HIV-1 gp120 V3 loop peptide sequences in complex with the corresponding receptors, CCR5 and CXCR4, based on the docked-and-minimized conformations which were used to produce the lowest binding free energy complex in [29,30]. The mutations were performed using CHARMM [36], and the initial conformation-orientation of the mutated side chains was preserved with regard to the initial conformation-orientation of the sidechains of [29,30].

All MD simulations and free energy calculations were performed using CHARMM. For each HIV-1 gp120 V3 loop:receptor complex, we performed two sets of MD simulations. The setup of the system and the parametrization used in both MD simulations sets were the same as that used in [29,30]. In the first set of MD simulations, we used the same equilibration and production run protocol that was used in [29,30]. In this study, the production run was equal to 4 ns. Upon the completion of the production run of each MD simulation, we performed a binding free energy analysis of the produced complex structures, and selected the lowest binding free energy complex structure per trajectory, which was provided as an input structure to the second set of MD simulations. In the second set of MD simulations, the selected structures from the first MD simulations were initially subjected to a slight equilibration prior to the production run. During the equilibration run, each complex structure was simulated for 200 ps during which the backbone-side chain atoms were constrained to their initial positions by a force constant equal to 1.0–0.4 kcal/mol*Å^2^. Subsequently, the entire complex was simulated for 100 ps with no constraints, so as to further relax the conformation of each complex structure prior to the final collection of structures in the production runs. During the production run, the complex structures were simulated for 5 ns, and snapshots were collected every 200 ps. For each HIV-1 gp120 V3 loop:receptor complex, the average residue pair wise interaction free energy for all interacting pairs was calculated using all twenty-five production run snapshots. The methodology used to calculate the residue pairwise interaction free energies was performed in a heterogeneous dielectric environment and is analytically described in references [29,30].

### Filtering of interacting pairs and interaction extraction

Simulations of CCR5 tropic sequences were considered separately from simulations of CXCR4 tropic sequences. Interacting pairs with median interaction energy of < -1 kcal/mol across the either 8 trajectories of CCR5 tropic sequences or 9 trajectories of CXCR4 tropic sequences were selected. Also, only interacting pairs involving amino acids of the V3-loop positions for which no amino acid is observed in more than 90% of the representative sequences were considered. Distances for each selected interacting residue pair were extracted from the lowest binding free energy snapshots of the first set of MD simulations for each of the representative sequences. Therefore, for each interacting pair from CCR5 we collected 8 distances and for each interacting residue pair from CXCR4 we collected 9 distances. The distances were converted to the distance bins that are present in the centroid to centroid distance bin force field that has be previously been described by our group [33].

Based on the selected interacting pairs and the extracted distance bin values, distance bin interaction energies were computed for each residue pair while using every sequence in our V3-loop superset. This was achieved by changing the V3-loop residues of the interacting pairs according to each sequence and recording the corresponding energy values for every observed distance bin. The minimum energy based on all possible distance bins is assigned for each interacting pair in each sequence. This resulted in an interaction table composed of 104 columns (one for each interacting pair) and 2455 rows (one for each V3-loop sequence). Additionally, the values for three established rules for coreceptor usage prediction (net charge, 11/24/25 rule, glycosylation motif) [9,12,13] were also computed for every sequence. We also propose a fourth rule, based on the length of the V3-loop, or the number of residues including the first and last cysteine, which is denoted as Rule IV and was also computed for every sequence.

### Interaction selection

In this work, the SVM model refers to the *l*^2^-norm formulation with kernel *K*(**x**_*i*_, **x**_*j*_). A well-known method for feature reduction utilizing SVMs with a linear kernel is the recursive feature elimination (RFE)-SVM algorithm[37], where at each iteration, the feature *k* with the lowest magnitude element $w_{k}^{2}$ of the weight vector **w** is eliminated from the feature basis. Usage of nonlinear kernels requires solving the dual formulation of the SVM model; in general, the weight vector cannot be calculated explicitly. The Hadamard product can be used to associate each instance vector **x**_*i*_ with a selection vector **z**:

$$x_{i}⟵x_{i}\circ z..$$

We propose the following criterion, based on the objective function of the dual formulation, which characterizes feature *k*’s importance in a given feature basis:

$${crit}_{k}=-\frac{1}{2}\sum_{i}\sum_{j}\alpha_{i}^{*}\alpha_{j}^{*}y_{i}y_{j}\frac{\partial K\left(x_{i}\circ z,x_{j}\circ z\right)}{\partial z_{k}}{|}_{z=1},$$

where $\alpha_{i}^{*},\alpha_{j}^{*}$ are the optimal values of the dual variables, *y*_*i*_, *y*_*j*_ are the class labels (parameters taking on the values of -1, 1), and **z** = **1** indicates *z*_*k*_ = 1, ∀*k*. Thus, the iterative algorithm for feature reduction is as follows:

$$\text{For each iteration},\;\text{remove feature}\;k^{*}={\text{arg max}}_{\text{k}}\{{crit}_{k}\}.$$

Note that this is equivalent to the RFE-SVM algorithm when performing linear classification, i.e., when *K*(**x**_*i*_, **x**_*j*_) = **x**_*i*_ ∙ **x**_*j*_. The algorithm has been implemented in Python/C using the libsvm library [38].

This nonlinear SVM feature selection was applied to the problem of interaction selection by starting with 104 interactions serving as the SVM feature basis, and iteratively removing one interaction at a time down to a single interaction. Since the interaction selection requires a training set as input, interaction selection was performed 100 times using 100 randomly selected training sets, which resulted in 100 sets of N interactions for N from 104 to 1. Consensus sets were created for a given number of interactions N, by taking the N interactions that were selected most often at that stage of the algorithm. Additional runs of interaction selection were performed while also including one or more established rules as features, but these additional features were never eliminated.

### SVM training and validation

Following interaction selection, the R [39] package e1071 [40] was used to train SVM models based on the Gaussian radial basis function using libsvm library for every identified feature set. In addition to a class prediction, the SVM models were trained to predict an associated probability for each prediction. Given a set of features 500 runs of 10-fold cross validation were performed based on the superset of V3-loop sequences. Based on the number of available CXCR4 tropic sequences, each test set contained 24 of each class, while the training sets contained 211 of each class. Since there are almost 10 times as many CCR5 tropic sequences as CXCR4 tropic sequences, for a given a selected set of CXCR4 tropic training samples, 10 sets of CCR5 tropic training samples were also selected and 10 SVM models trained. This was performed in order to take full advantage of all available data, and therefore, for each feature set 50,000 SVMs were trained and the average of the probabilities of the 10 SVM models was utilized. During the cross-validation runs, the accuracy of feature sets was evaluated generating receiver operator curves calculated within R and the area under the curve (AUC) was calculated using the pracma R package [41]. Additional metrics of accuracy were also computed including the classification accuracy, calculated as the percent of the samples for which the coreceptor usage was predicted correctly, and the sensitivity at a false positive rate (FPR) of 0.05%.

### Comparison with other methods

In order to compare CRUSH to existing methods, we split our 2455 V3-loop sequences into a training set, consisting of the 1294 sequences that were found in Dybowski et al. [23], referred as the Dybowski set, and a test set consisting of the remaining 1161 sequences. To avoid bias, a second model based on the 15 features of the all rules model was trained using the Dybowski set. However, the Dybowski set only contains about 7 times as many CCR5 tropic sequences as CXCR4 tropic sequences, therefore 7 SVM models were trained rather than the 10 SVM models used for cross validation. We contacted the developers of geno2pheno to compare our datasets in order to perform an unbiased comparison, but they declined to provide the data. Instead we have trained a model based on a binary representation of aligned V3-loop sequences, referred as g2p, as described by Bozek et al. [24]. g2p has been used previously by the authors of geno2pheno as an alternative for comparing to geno2pheno[coreceptor] due to differences in training sets. Our implementation of g2p involved first aligning all 2455 V3-loop sequences, plus the V3-loop sequence from PDB structure 2B4C [20], using MAFFT [42]. The resulting multiple sequence alignment had a width of 57, which resulted in 1140 total binary features that were used to train an SVM model as described for CRUSH above. The reduced CRUSH model, T-CUP2 [43], the probit method of Kieslich et al. [9], and g2p [24] were applied to the 1161 sequence test set. The T-CUP2 R package was used in testing, while R code was written to compute probit probabilities according to Table 5 of Kieslich et al. [9]. Additionally, a similar benchmark was also performed using the unique patient subset by training a CRUSH model on the 708 sequences (625 CCR5/ 83 X4) of the unique patient subset that were found in the Dybowski set and testing the methods on the remaining 373 sequences (342 CCR5/ 31 CXCR4).

To test accuracy across the major HIV-1 subtypes, additional subtype specific test sets were extracted from the 1161 sequence test set described above. The 1161 sequences contained both CCR5-specific and CXCR4-specific sequences for four major subtypes, A/AG, AE, B, and C (as defined in [18]), resulting in four subtype specific test sets. The sequences of these four major subtypes compose over 75 percent of the 1161 sequences, while the remaining sequences belong to minor subtypes or have been assigned to multiple subtypes. For comparison, the reduced CRUSH model and PhenoSeq [18] subtype specific models were applied to each of the subtype specific test sets. Subtype specific test sets were also derived from the unique patient subset for further validation.

## Results

We initially modeled the structures of representative HIV-1 gp120 V3-loop peptide sequences (8 CCR5 tropic and 9 CXCR4 tropic) in complex with the corresponding receptors based on complex structures from [29,30]. Molecular dynamics simulations and free energy calculations were performed so as to improve the conformational properties of each HIV-1 gp120 V3-loop sequence in complex with each receptor, and to investigate which are the critical interactions formed between different HIV-1 gp120 V3-loop sequences in complex with the two receptors. To identify interacting residue pairs important for V3-loop binding to CCR5 and CXCR4, simulations of CCR5 tropic sequences were considered separately from simulations of CXCR4 tropic sequences. To reduce the number of residue pairs, only interacting pairs with median interaction energy of < -1 kcal/mol across the either 8 trajectories of CCR5 tropic sequences or 9 trajectories of CXCR4 tropic sequences were considered in subsequent modeling. According to our molecular dynamics simulations and filtering criteria we identified 52 interacting residue pairs for CCR5 and 52 interacting residue pairs for CXCR4 (Tables A and B in S1 Tables). We collected residue pair distances from our molecular dynamics simulations, and computed the minimum centroid-centroid energies for every interacting pair based on every V3-loop sequence of our superset.

**Fig 2:**
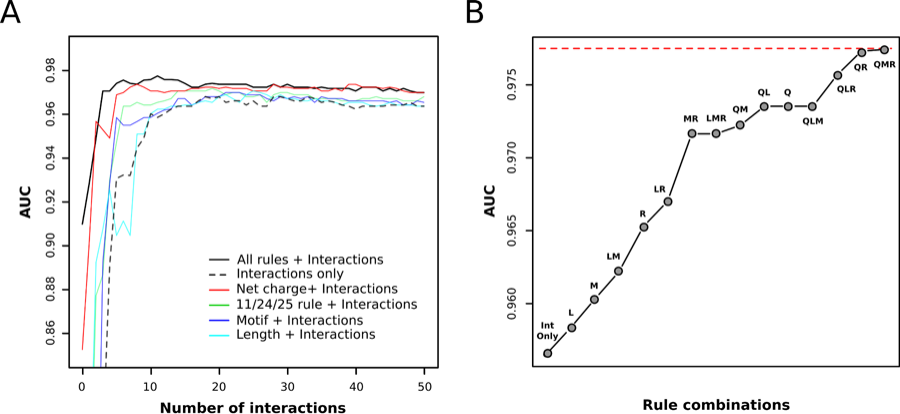
Effect of the rules and V3-loop:coreceptor interactions included in SVM models on prediction accuracy. (A) The effect of the number of V3-loop:coreceptor interactions on accuracy. Accuracy is represented by the median AUC for 500 runs of 10-fold cross validation for both panels A and B. The accuracy at zero interactions is the accuracy based on only the rules. (B) Contribution of rules to accuracy when used in addition to the top 11 interactions Fig 3A. The naming scheme is as follows: Int Only–interactions only; Q–net charge; R – 11/24/25 rule; M–glycosylation motif; L–length. Dashed red line illustrates the accuracy when using all four rules and the top 11 interactions (QLMR, 0.977).

A novel nonlinear feature selection algorithm was applied to the problem of interacting residue pair selection by starting with 104 interactions serving as the SVM feature basis, and iteratively removing one interaction at a time down to a single interaction. To investigate the contribution of the rules (Net charge, 11/24/25, motif, length) to coreceptor usage prediction, interaction selection was performed six times while also including one or more rules as features that were not considered for removal (Fig 2A). Given a set of interactions and/or rules, 500 runs of 10-fold cross validation were performed based on a superset of V3-loop sequences, resulting in over 30 million SVMs being trained in total for this study.

During the cross-validation runs, the accuracy of feature sets was evaluated by calculating the area under the receiver operator curve (AUC), where a value of 1 represents a perfect classification. Additionally, the sensitivity at a false positive rate of 0.05 was also calculated. Of the 624 sets of interactions/rules that were evaluated for predictive utility, the best model in terms of accuracy with the fewest features is summarized in Table 1. Our model utilizes only 15 total features (11 interactions and 4 rules), while other existing methods have hundreds of features [24], which is significant when considering that balanced training sets contain only ~400 samples.

**Table 1:** Summary of best coreceptor usage model.

Number of interacting residue pairs	11
Additional rules	Net charge, 11/24/25 rule, Motif, Length
Median AUC^a^	0.977
Median sensitivity at 0.05 FPR^a^	0.917

^a^ Results based on 500 runs of 10-fold cross-validation.

**Fig 3:**
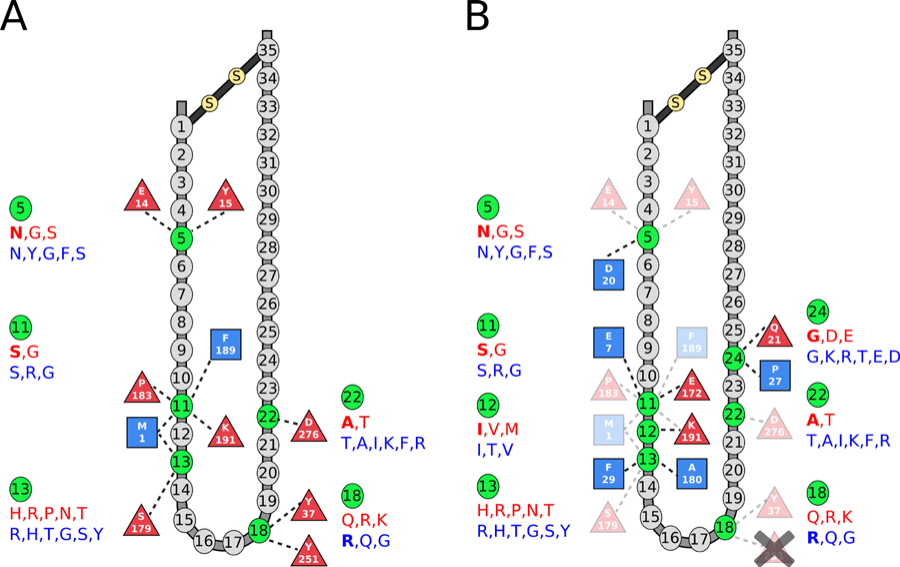
Diagrams of the top selected interactions for the cases of all rules + interactions and interactions only. **(**A) Interaction map for the 11 interactions selected in combination with all rules. V3-loop is shown as an idealized loop with 35 amino acids where grey circles indicate positions for which no interactions were selected (inactive), while green circles indicate V3-loop positions with interactions selected (active). Red triangles represent residues of CCR5 and blue squares represent residues of CXCR4, with dashed lines representing interactions with V3-loop residues. Ordered lists of observed amino acids (based on occurrence with a minimum of 5%) in one-letter code for each active V3-loop residue are provided. Observed amino acids for CCR5 tropic sequences are in red and those observed for CXCR4 tropic sequences in blue. Bolded letters in the ordered list of observed amino acids indicate an amino acid that is observed in at least 50% of sequences at a given position. (B) Interaction map for the 18 interactions selected without rules. Color scheme and layout is the same as in (A). Faded triangles/squares indicate interactions that were also selected when including all rules. The crossed out interaction was selected when including rules, but not when using interactions only.

The identified interacting residue pairs alone provide highly accurate predictions (Fig 2A, dashed line), with a maximum interactions only accuracy of 0.969 being achieved for a set of 18 interactions (Fig 3B). This result is evidence for the validity of the selected interacting residue pairs and the computational models from which they were derived [29,30]. As is illustrated by Fig 2A, it is clear that fewer interactions are needed to obtain a high level of accuracy when using the rules as additional features, since the rules only model (Fig 2A, zero interactions) provides an AUC of 0.910. Of the four evaluated rules, net charge was the only rule to perform as well as all rules, but only once additional interactions were added. Electrostatically driven protein association has been proposed to be composed of two steps: recognition, which results in an initial encounter complex and is driven by long range electrostatic interactions; binding, which involves short and medium range interactions (both polar and nonpolar), and results in a specific bound complex [44]. Therefore, the absence of rules that capture aspects of the binding step can be over come by introducing additional interactions, while rules inferring details of recognition may not be fully captured by adding additional interactions. Net charge captures the global electrostatic characteristics of the V3-loop that drive the recognition stage of association, and as a result additional interactions are not able to account for absence of net charge as a feature. The contributions of each of the rules is further illustrated by Fig 2B based on the accuracy of all possible combinations of the rules with the same 11 interactions (Fig 3A). Net charge (Q) outperformed all combinations in which net charge was not used, including four combinations of multiple rules.

For the interactions only model, interactions with two additional V3-loop position not found in all rules case was added, positions 12 and 24. The interactions with position 24 most likely help accommodate for the absence of the 11/24/25 rule. The interaction with position 12 involves Lys 191 of CCR5, which also participates in an interaction with V3-loop position 11. Interestingly, position 12 is often hydrophobic in CCR5 and CXCR4 tropic sequences, while position 11 tends to be occupied by a polar amino acid (Fig 3). One interaction selected for the all rules case was not selected for the interactions only model, V3-loop residue 18 with CCR5 Tyr 251.

Of the interactions identified by this study (Fig 3), thirteen have been previously associated with HIV-1 coreceptor activity by experiments summarized in [29,30], and four additional residue pairs are only one position away from an experimentally identified interaction. However, three of the CXCR4 interactions identified in this study (V3 13 –CXCR4 Phe 29; V3 13 –CXCR4 Ala 180; V3 24 –CXCR4 Pro 27) have not been previously suggested to contribute to V3-loop binding. The participating residues were in proximity in the previously predicted structures, but did not meet the energy threshold to grant mentioning [29,30]. These three interactions were most likely identified in this study since we considered multiple representative R5- and X4-tropic sequences in complex with CCR5 or CXCR4.

In this study, V3-loop position 11 is found to be most important for determining coreceptor selection, with four selected interactions in the all rules+interactions model (Fig 3A) and an additional two interactions selected in the interactions only model (Fig 3B). Positions 5, 13 and 18 all are involved in two interactions selected in the all rules model, while position 13 is involved in an additional two interactions in the interactions only model. V3-loop position 24 is also involved in two interactions, but only in the interactions only model. Sander et al. [22] also identified V3-loop residues 11,13, 22, and 24 (denoted as 306, 308, 317, and 319) as being important for coreceptor usage. Dybowski et al. [23] also points to V3-loop position 11 as being key to tropism, while of the residues identified by Bozek et al. [24] only postions 12 and 24, which were identified by the interactions only model, overlap with the residues identified by this study.

Based on the all rules model we have developed a web tool named CoReceptor USage prediction for HIV-1 (CRUSH). The implementation is very efficient, since CRUSH does not require expensive calculations, such as molecular dynamics simulations, not even a sequence alignment, and all features are computed by either simple character counting, for the case of the four rules, or by a table lookup, for the eleven interactions (see S1 Text for more details). In order to compare CRUSH to existing methods we trained an additional model based on the 15 features of the all rules model using only the 1294 V3-loop sequences from the superset that were also used by Dybowski et al. [23] (see Methods). The remaining 1161 sequences in our superset were used as a test set to compare three recent methods T-CUP2 [43], probit [9], and g2p [24], which were selected since the source code is available or could be implemented locally, allowing for testing on such a large test set. Tables 2 and 3 summarize the method comparison.

**Table 2:** Comparison of methods on test set (1161 sequences).

Method	Sensitivity at FPR 0.02	Sensitivity at FPR 0.05	Number of features	Total test CPU time (sec)
CRUSH	0.797	0.892	15	0.59
Probit	0.723	0.811	3	0.13
T-CUP2	0.635	0.770	70	222.46
g2p*	0.486	0.676	1140	-

* The g2p method as described by Bozek et al. [24] requires that a sequence alignment be performed on the entire dataset prior to training/testing preventing an equal comparison of CPU time.

**Table 3:** Method accuracy comparison on unique patient test set (373 sequences).

Method	Sensitivity at FPR 0.02	Sensitivity at FPR 0.05
CRUSH	0.677	0.839
probit	0.645	0.742
T-CUP2	0.581	0.774
g2p	0.161	0.258

On the test set, CRUSH provides at least a 10% improvement in sensitivity, at a FPR of 0.02 or 0.05, when compared to all three methods (Table 2). The accuracy of CRUSH is reduced when using the unique patient subset (Table 3), but the accuracies observed for the competing methods are also reduced, resulting in the ranking of methods being the same when considering either one or multiple sequences per patient. Previously reported accuracies for T-CUP2 [43] and g2p [24] are comparable to those obtained on our test set. The accuracy of the CRUSH web tool is expected to be even higher, since the model used for method comparison was trained on about half as many V3-loop sequences. All methods were tested on the same computer using R [39] implementations, and the total computational times required by each method to make predictions for the entire test set are included in Table 2. CRUSH is almost 400 times faster than T-CUP2, which is actually an improved implementation of the method developed by Dybowski et al. [23]. CRUSH is also substantially more efficient than the method developed by Bozek et al. [24], whose web server warns that the computational time for each sequence could be as much as 30 seconds. The speed of the probit method, however, is comparable to CRUSH, which is due to the fact that the probit prediction is simply a table lookup given the values for net charge, the glycosylation motif, and 11/24/25 rule, while CRUSH requires a function evaluation given the values of the 15 features.

In recent years, an area of significant interest has been the use of next generation sequencing, which could produce well over 1 million reads per sample, to predict HIV-1 coreceptor usage [45–48]. Therefore, computationally efficient methods for HIV-1 coreceptor prediction, capable of analyzing very large datasets, are needed. The authors of T-CUP2 have recently released, gCUP [45], an implementation of T-CUP2 optimized for GPUs, that was developed for use with next generation sequencing data and is able to make predictions for over 175,000 sequences per second using GPUs. Given that gCUP is an optimized implementation of T-CUP2, and CRUSH is significantly more efficient than T-CUP2, an optimized/parallel implementation of CRUSH could definitely be applied to next generation sequencing analysis and should be the focus of future work.

Another important consideration for genotypic prediction of HIV-1 coreceptor usage is whether a method can provide accurate predictions across the major HIV-1 subtypes, since many methods require an alignment to a reference sequence and have been developed for HIV-1 subtype B. To evaluate the accuracy of CRUSH on different HIV-1 subtypes we have decomposed our test set into four subtype specific test sets (Tables 4 and 5), and utilized the reduced CRUSH model to predict coreceptor usage. For comparison, the suite of subtype specific models, PhenoSeq [18], was also applied to the subtype specific test sets and results are summarized by Tables 4 and 5. The PhenoSeq suite does not provide a prediction score, only a classification; therefore, the AUC values reported for PhenoSeq are calculated using three-point ROC curves based on the specificity and sensitivity of the classification. Additionally, the reported sensitivity for CRUSH in Tables 4 and 5 is the sensitivity achieved by CRUSH given the corresponding specificity of PhenoSeq for each test set.

**Table 4:** Comparison of HIV-1 subtype accuracies for test set.

Subtype *(# X4/# R5)*	CRUSH AUC	CRUSH Sensitivity*	PhenoSeq AUC	PhenoSeq Sensitivity	PhenoSeq Specificity
A/AG *(4/65)*	0.758	0.750	0.721	0.750	0.692
AE *(6/53)*	0.994	1.000	0.981	1.000	0.962
B *(29/490)*	0.957	0.931	0.861	0.966	0.757
C *(13/227)*	0.987	1.000	0.899	1.000	0.797
Total *(52/876)*	0.953	0.942	0.869	0.962	0.777

* CRUSH sensitivity is based on a probability threshold that produces the corresponding PhenoSeq specificity.

**Table 5:** Comparison of HIV-1 subtype accuracies for unique patient test set.

Subtype *(# X4/# R5)*	CRUSH AUC	CRUSH Sensitivity*	PhenoSeq AUC	PhenoSeq Sensitivity	PhenoSeq Specificity
A/AG *(4/33)*	0.758	0.750	0.784	0.750	0.818
AE *(1/25)*	1.000	1.000	0.980	1.000	0.960
B *(4/95)*	0.889	0.750	0.754	0.750	0.758
C *(9/59)*	1.000	1.000	0.915	1.000	0.831
Total *(18/233)*	0.922	0.889	0.852	0.889	0.815

* CRUSH sensitivity is based on a probability threshold that produces the corresponding PhenoSeq specificity.

Table 4 shows that CRUSH provides AUC accuracy of greater than 0.95 overall, as well as for three of the four subtype specific test sets, while producing higher AUC accuracies than PhenoSeq for all four subtype sets. For subtype B (Table 4), as well as for the total set, PhenoSeq produces a higher sensitivity than CRUSH, but at very poor specificity values of 0.757 and 0.777, respectively. To put this into context, a specificity of 0.757 or 0.777 implies that over 20% of CCR5 sequences are misclassified as CXCR4, and therefore hypothetically would be falsely excluded from CCR5 antagonist treatment. Since PhenoSeq only provides a classification and not a score, PhenoSeq cannot be tuned to provide a specificity that falls into the range 0.95 to 0.98 that is typically used when evaluating HIV-1 coreceptor prediction.

Based on the unique patient subset (Table 5), the sensitivity of CRUSH equals that of PhenoSeq for every subtype, as well as for the test set as a whole. However, Table 5 shows that CRUSH provides a 7% improvement in the total AUC accuracy for the unique patient subset, and also produces a higher AUC accuracy for every subtype except A/AG. The subtype specific accuracies observed for the test sets containing multiple sequences per patient (Table 4) and one sequence per patient (Table 5) are similar, and both illustrate the accuracy of CRUSH across subtypes. CRUSH provides comparable accuracy across major subtypes because it does not utilize sequence alignments to template sequences and therefore is not limited to specific subtypes.

At present time, the CRUSH server has yet to be validated in a clinical setting, which is to be the focus of future studies. In the current implementation, the CRUSH server provides a probability score for each submitted V3-loop sequence, but clinically relevant thresholds have yet to be established. For the test set results summarized in Table 2, a probability threshold of ~0.90 provided a specificity of 0.98 while a probability of ~0.75 provided a specificity of 0.95. These probability cutoffs could be used as a rule of thumb until clinically relevant thresholds can be established.

In summary, we have identified a finite set of eleven interactions that can be used to accurately predict coreceptor usage. The manner in which the mutations affect the interactions with the coreceptors is highly nonlinear, since sequences specific to CCR5 or CXCR4 could contain the same amino acids at some positions. Our proposed nonlinear SVM models, made available through the CRUSH server, are able to decipher these complex relationships, providing highly accurate predictions of coreceptor usage with potential utility for clinical settings.

## Supporting Information

S1 Dataset Superset of non-redundant HIV-1 V3-loop sequences. (TXT)

S2 Dataset Unique patient subset of HIV-1 V3-loop sequences. (TXT)

S1 Tables CCR5:V3-loop and CXCR4:V3-loop interacting residue pairs. (PDF)

S1 Text CRUSH procedure outline. (PDF)
