# Supplementary material for: Highly Accurate Structure-Based Prediction of HIV-1 Coreceptor Usage Suggests Intermolecular Interactions Driving Tropism
Source: PLoS One. 2016 Feb 9;11(2):e0148974. doi: 10.1371/journal.pone.0148974 (PMC4747591; doi:10.1371/journal.pone.0148974)
Supplement: S1 Tables — (PDF) [file pone.0148974.s003.pdf]

Table A. Filtered 52 interacting residue pairs for CCR5 tropic sequences

V3 residue	R5 residue	Median Energy[a]	Mean Distance[b]	Distance bins[c]
2	15	-1.06	9.21	3-1:7-1:8-2:9-4
5	14	-4.32	6.56	3-1:4-3:5-2:6-1:7-1
5	15	-2.37	7.17	3-2:4-1:5-1:7-1:8-2:9-1
10	17	-4.81	5.46	1-4:2-1:5-1:6-2
10	20	-1.46	6.56	1-2:2-2:3-1:7-1:9-2
10	188	-1.86	7.21	1-1:2-1:5-1:8-5
10	191	-3.54	8.03	5-1:6-2:7-1:8-3:9-1
10	261	-4.10	6.34	1-1:2-2:3-1:5-2:6-1:8-1
10	262	-7.09	5.98	1-1:3-5:6-2
10	264	-2.89	5.53	1-2:2-2:3-2:4-1:5-1
10	272	-2.41	6.57	2-2:3-2:6-2:7-1:8-1
11	172	-1.34	6.08	1-3:3-1:4-1:6-1:7-1:9-1
11	183	-1.38	5.55	1-1:2-3:3-3:5-1
11	191	-1.35	6.40	1-1:3-1:4-1:5-4:8-1
12	179	-1.70	7.67	5-1:6-4:7-2:9-1
12	180	-3.43	6.35	1-2:2-2:3-1:4-1:8-1:9-1
12	191	-2.06	6.69	3-1:4-4:6-2:8-1
13	172	-3.37	5.49	1-1:2-4:3-1:5-2
13	177	-1.25	5.41	1-3:2-2:3-1:4-1:6-1
13	178	-3.33	8.78	7-1:8-3:9-4
13	179	-2.41	4.66	1-5:2-1:3-1:4-1
14	86	-1.05	6.85	4-2:5-3:6-3
14	89	-3.52	5.67	1-1:2-1:3-4:4-2
14	177	-3.05	6.84	3-1:4-1:5-3:6-2:7-1
14	178	-5.22	5.34	1-1:2-5:3-2
14	179	-1.10	7.91	6-2:7-3:8-3
18	37	1.39	6.22	3-1:4-6:5-1
18	79	-1.22	6.07	3-4:4-4
18	86	-4.86	5.25	1-2:2-2:3-4
18	108	-7.47	5.43	2-6:3-1:4-1
18	248	-1.43	7.57	6-4:7-2:8-2
18	251	-6.44	4.94	1-4:2-3:3-1
18	279	-1.39	8.50	8-7:9-1
18	283	-70.85	4.24	1-8
18	286	-9.06	7.26	4-1:5-2:6-3:7-1:8-1
18	289	-11.49	7.58	4-2:5-2:6-1:9-3
19	251	-1.22	7.21	5-3:6-3:7-1:8-1
19	279	-2.77	4.38	1-7:2-1
19	280	-1.50	5.33	1-3:2-3:4-2
19	283	-1.77	5.10	1-3:2-4:3-1
20	251	-1.74	6.95	5-5:6-2:8-1
20	255	-1.25	5.86	1-1:2-2:3-1:4-2:5-2
20	276	-1.16	9.57	8-3:9-5
20	279	-3.09	6.16	1-1:3-1:4-2:5-4
22	272	-1.95	4.67	1-6:2-1:5-1
22	276	-8.41	4.71	1-7:3-1
24	20	-2.79	11.76	9-8
24	21	-3.01	11.35	9-8
24	172	-1.58	6.84	1-1:2-1:4-2:6-1:7-1:8-2
25	21	-2.70	14.00	9-8
27	1	-1.71	7.99	1-2:4-1:5-1:8-1:9-3
27	3	-1.31	8.66	4-1:5-2:8-1:9-4

a – Median interaction energy for each residue pair across the selected snap shots of the eight V3-loop:CCR5 complexes
b – Mean distance for each residue pair across the selected snap shots of the eight V3-loop:CCR5 complexes
c – Observed distance bins across the selected snap shots of the eight V3-loop:CCR5 complexes (bin #-count)
d – Light blue indicates pairs selected when including interactions+rules; dark blue indicates pairs selected when including interactions only

Table B. Filtered 52 interacting residue pairs for CXCR4 tropic sequences

V3 residue	X4 residue	Median Energy[a]	Mean Distance[b]	Distance bins[c]
5	20	-2.01	5.87	1-2:2-2:3-2:4-1:6-1:9-1
5	21	-1.34	11.28	9-9
5	22	-1.68	8.21	3-1:4-1:5-1:6-1:7-1:8-1:9-3
5	23	-3.27	7.08	1-2:2-1:3-1:4-1:8-1:9-3
10	12	-9.94	5.65	1-3:3-3:5-2:6-1
10	20	-1.65	8.70	4-1:5-1:8-4:9-3
10	21	-1.05	9.26	7-1:8-3:9-5
10	193	-3.85	4.72	1-4:2-4:3-1
11	1	-1.61	13.55	9-9
11	7	-7.25	11.44	1-1:9-8
11	189	-1.24	7.20	2-1:3-1:4-2:5-1:6-1:7-1:8-1:9-1
12	189	-2.67	5.77	1-2:2-2:3-1:4-3:5-1
12	190	-2.83	6.20	1-2:2-2:4-1:5-1:6-3
12	196	-1.39	5.86	1-2:3-4:5-3
13	1	-2.04	14.75	4-1:9-8
13	29	-2.19	6.11	1-1:2-1:3-2:4-2:5-1:6-1:7-1
13	30	-1.49	7.19	3-2:4-1:6-1:7-4:9-1
13	180	-1.06	5.54	1-5:3-1:4-1:5-1:8-1
13	181	-1.25	7.15	2-3:3-2:6-1:8-2:9-1
14	30	-1.98	7.76	1-2:3-1:6-1:7-1:8-1:9-3
14	180	-2.42	4.72	1-8:5-1
14	183	-1.78	8.26	3-2:4-1:5-2:9-4
14	185	-1.77	6.18	1-1:3-3:4-2:5-2:7-1
14	187	-5.50	5.10	1-6:4-2:6-1
14	189	-1.03	8.02	3-1:4-2:6-1:7-1:8-1:9-3
18	113	-3.42	6.08	1-1:2-1:3-2:4-2:5-2:6-1
18	117	-6.82	5.06	1-4:2-4:3-1
18	171	-17.73	5.99	1-1:3-2:4-5:5-1
18	188	5.68	5.19	1-4:2-3:3-1:4-1
18	203	-3.12	5.85	1-1:2-1:3-3:4-4
18	288	-60.49	4.81	1-6:2-2:4-1
19	281	-1.99	5.43	1-2:2-3:3-2:4-1:5-1
19	285	-1.14	4.60	1-8:4-1
19	288	-3.19	5.64	1-2:2-3:3-1:4-1:5-1:6-1
20	188	-2.43	6.31	1-1:2-3:4-1:5-1:6-1:8-2
20	190	-1.89	5.89	1-1:2-2:3-1:4-3:5-2
20	196	-1.60	5.32	1-2:2-4:3-1:4-2
20	199	-1.35	8.19	5-2:6-2:7-2:9-3
20	200	-3.86	5.22	1-6:5-2:6-1
20	259	-1.27	6.97	2-1:3-3:4-1:5-1:6-1:9-2
20	262	-1.65	6.60	1-1:2-1:3-1:5-2:6-3:8-1
20	281	-2.39	9.06	6-1:7-1:8-1:9-6
20	284	-1.39	7.37	3-2:4-1:5-2:8-3:9-1
22	193	-9.82	5.81	1-5:6-2:7-1:8-1
22	266	-2.21	5.03	1-5:2-1:3-1:4-2
22	277	-1.15	6.72	1-1:5-4:6-3:8-1
24	25	-3.61	5.74	1-2:2-2:3-3:4-1:9-1
24	27	-1.07	7.08	1-1:2-1:3-2:4-1:5-1:9-3
25	1	-1.73	11.84	9-9
25	25	-2.07	9.68	6-1:8-1:9-7
25	27	-2.33	5.80	1-1:2-2:3-3:4-2:6-1
27	12	-1.59	15.34	9-9

a – Median interaction energy for each residue pair across the selected snap shots of the nine V3-loop:CXCR4 complexes
b – Mean distance for each residue pair across the selected snap shots of the nine V3-loop:CXCR4 complexes
c – Observed distance bins across the selected snap shots of the nine V3-loop:CXCR4 complexes (bin #-count)
d – Light blue indicates pairs selected when including interactions+rules; dark blue indicates pairs selected when including interactions only
