# Supplementary material for: Highly Accurate Structure-Based Prediction of HIV-1 Coreceptor Usage Suggests Intermolecular Interactions Driving Tropism
Source: PLoS One. 2016 Feb 9;11(2):e0148974. doi: 10.1371/journal.pone.0148974 (PMC4747591; doi:10.1371/journal.pone.0148974)
Supplement: S1 Text — (PDF) [file pone.0148974.s004.pdf]

CRUSH procedure outline

1. The CRUSH web server takes as input V3-loop sequences (as defined by the first and last cysteine) in FASTA format. For all rules and interactions, V3-loop position 1 refers to the first cysteine residue, and positions increase one at a time from there without a reference alignment.
2. Subsequently the 15 features are extracted from the sequence(s), namely: net charge, glycosylation motif (binary), 11/24/25 rule (binary), length, and 11 V3-loop:coreceptor interaction energies.

    - Net charge is computed by simply summing the number of K/R and subtracting the number of D/E.
    - If the V3 loop has N at position 6, not a P at position 7, S or T at position 8, and not a P at position 9 a value of 1 is assigned for glycosylation motif, while 0 is assigned otherwise.
    - If at least one K or R is located at positions 11, 24, or 25 a value of 1 is assigned to 11/24/25 rule, and 0 is assigned otherwise.
    - Length is simply the number of amino acids in the V3-loop sequence including the first and last cysteine
    - The 11 interaction energies (Table S3) are assigned using lookup tables that are included below. The first column is used to identify the appropriate row depending on the amino acid found at the position number in the parentheses, while the remaining columns contain energy values for different observed distance bins. After the appropriate row is identified for a given interaction, the minimum energy across the observed bins is assigned.

3. The extracted features are put into a table with 15 columns, one for each feature, and where each row corresponds to an uploaded FASTA sequence.
4. The table of features is then used as input for the R predict() function, which outputs the predicted X4 probability based on the input feature table (step 3) and SVM models that were trained based on the V3-loop sequence superset using the svm() function of the R library e1071.
5. As described in Methods, CRUSH utilizes the average probability from 10 separate SVM models that were trained using the same CXCR4 sequences, but different CCR5 sequences.

Top 11 selected interactions

#	V3 residue	Coreceptor	Residue	Amino acid	Bins
1	5	CCR5	14	E	3,4,5,6,7
2	5	CCR5	15	Y	3,4,5,7,8,9
3	11	CCR5	183	P	1,2,3,5
4	11	CCR5	191	K	1,3,4,5,8
5	11	CXCR4	1	M	9
6	11	CXCR4	189	F	2,3,4,5,6,7,8,9
7	13	CCR5	179	S	1,2,3,4
8	13	CXCR4	1	M	4,9
9	18	CCR5	37	Y	3,4,5
10	18	CCR5	251	Y	1,2,3
11	22	CCR5	276	D	1,3

Interaction #1

V3 (5)	bin3	bin4	bin5	bin6	bin7
A	0.00071	-0.00036	-0.00013	0.00065	0.00010
C	-0.00117	-0.00039	-0.00021	-0.00004	-0.00026
D	-0.0006	-0.00076	-0.00122	-0.0003	-0.00007
E	-0.00038	-0.00007	-0.00044	-0.00047	0.00004
F	-0.00084	-0.00027	-0.00083	-0.00054	-0.00096
G	-0.00052	0.00018	-0.00017	0.00014	0.00041
H	0.00017	-0.00044	-0.00066	-0.00028	-0.00029
I	-0.00074	-0.0003	0.00045	0.00022	0.00013
K	-0.00015	-0.0009	-0.00038	-0.00058	0.00047
L	0.00018	0.00028	-0.0001	0.00032	0.00030
M	-0.0002	-0.0002	-0.00019	0.00005	-0.00012
N	-0.00126	-0.00048	-0.00075	-0.00015	-0.00023
P	0.00016	-0.00046	0.00025	-0.0002	-0.00038
Q	-0.00076	-0.00048	0.00015	-0.00023	0.00012
R	0.00007	-0.00002	-0.00029	-0.00046	0.00010
S	0.00006	-0.00087	-0.0002	0.00015	0.00026
T	-0.00027	0.00008	-0.00037	-0.00044	-0.00041
V	-0.0006	-0.00129	-0.00072	-0.00027	0.00030
W	0.00011	-0.00099	-0.00029	0.00024	0.00013
Y	0.00071	0.00058	0.00049	-0.00001	0.00033

Interaction #2

V3 (5)	bin3	bin4	bin5	bin7	bin8	bin9
A	0.00045	-0.0002	-0.00006	-0.00042	0.00009	0.00009
C	-0.00076	0.00025	0.00058	0.00001	0.00015	0.00015
D	-0.00006	0.00011	0.00016	-0.0006	-0.00009	-0.00009
E	0.00071	0.00058	0.00049	0.00033	0.00034	0.00034
F	0.00000	-0.00057	-0.00047	-0.0013	-0.00012	-0.00012
G	-0.00126	-0.00088	-0.00055	0.00009	-0.00056	-0.00056
H	0.00018	0.00064	0.00057	0.00038	-0.00012	-0.00012
I	0.00066	0.00083	0.00026	0.00029	0.00022	0.00022
K	0.00111	0.00124	0.00053	0.00061	0.00022	0.00022
L	-0.0009	-0.00061	-0.00056	-0.00023	-0.00039	-0.00039
M	-0.0008	-0.00038	-0.00011	0.00016	0.00020	0.00020
N	-0.00005	0.00009	0.00033	0.00011	0.00004	0.00004
P	-0.00084	0.00010	0.00004	-0.00045	0.00004	0.00004
Q	-0.0011	-0.00077	-0.00024	-0.00047	-0.00051	-0.00051
R	0.00099	0.00136	0.00079	0.00067	0.00048	0.00048
S	0.00008	-0.00042	0.00044	0.00023	0.00004	0.00004
T	0.00095	0.00084	0.00085	0.00090	0.00072	0.00072
V	-0.00141	-0.00014	-0.00007	-0.00049	-0.00061	-0.00061
W	-0.00077	-0.00049	-0.00015	-0.00041	0.00008	0.00008
Y	-0.0009	-0.00078	-0.0002	0.00001	-0.00007	-0.00007

Interaction #3

V3 (11)	bin1	bin2	bin3	bin5
A	0.00050	-0.00008	0.00076	0.00046
C	-0.00164	-0.00006	-0.00147	0.00080
D	-0.00134	-0.00082	0.00064	0.00063
E	-0.0005	0.00020	0.00016	0.00025
F	0.00031	0.00086	-0.00112	-0.00093
G	-0.00339	-0.00199	-0.0008	-0.00158
H	-0.00042	0.00012	0.00072	0.00130
I	-0.00016	-0.0005	-0.0003	-0.00049
K	-0.00075	-0.0011	-0.0007	-0.00005
L	-0.00028	0.00052	-0.00099	-0.00078
M	-0.00186	-0.00139	-0.001	-0.00018
N	-0.00174	-0.00062	-0.00042	0.00084
P	-0.00014	0.00101	0.00228	0.00162
Q	-0.00235	-0.00238	-0.00234	-0.00036
R	0.00073	0.00132	-0.00021	-0.00018
S	-0.00131	0.00131	-0.00031	-0.00052
T	0.00006	0.00106	0.00067	0.00072
V	-0.00252	0.00026	0.00059	0.00138
W	0.00087	0.00010	-0.00083	0.00008
Y	-0.0006	-0.00037	-0.00084	0.00004

Interaction #4

V3 (11)	bin1	bin3	bin4	bin5	bin8
A	0.00005	0.00010	0.00029	0.00001	-0.00019
C	0.00020	-0.0007	0.00031	-0.00132	0.00057
D	0.00040	0.00082	0.00144	0.00077	-0.00012
E	-0.00052	-0.00015	-0.0009	-0.00038	-0.00058
F	-0.00221	-0.00144	-0.00151	-0.00176	-0.00085
G	-0.0003	-0.00083	0.00045	0.00074	0.00011
H	-0.00166	0.00021	0.00145	-0.00008	0.00053
I	-0.00144	-0.00075	-0.00024	-0.00064	0.00025
K	-0.00214	-0.00215	0.00030	0.00114	-0.00029
L	-0.00038	-0.0004	-0.00115	-0.00008	-0.00003
M	-0.00145	0.00061	0.00001	-0.0001	-0.00015
N	-0.00033	-0.00087	-0.00068	-0.00124	0.00006
P	-0.00075	-0.0007	0.00025	-0.00005	-0.0004
Q	-0.00048	-0.00098	-0.00039	0.00014	0.00046
R	-0.00194	-0.00015	0.00072	0.00045	-0.00023
S	-0.00093	0.00037	0.00001	0.00103	0.00027
T	-0.00052	-0.00032	-0.00015	-0.00061	0.00036
V	0.00021	0.00032	-0.00075	-0.00085	-0.00016
W	-0.00263	-0.00057	-0.00137	-0.00002	0.00003
Y	-0.00063	0.00111	0.00124	0.00053	0.00022

Interaction #5

V3 (11)	bin9
A	0.00018
C	-0.00003
D	-0.00022
E	-0.00018
F	-0.0005
G	0.00015
H	-0.00051
I	-0.00021
K	-0.00015
L	-0.00042
M	-0.00025
N	-0.00033
P	-0.0003
Q	0.00011
R	0.00023
S	-0.00001
T	0.00063
V	0.00027
W	0.00020
Y	0.00020

Interaction #6

V3 (11)	bin2	bin3	bin4	bin5	bin6	bin7	bin8	bin9
A	-0.00067	0.00000	-0.00058	-0.0007	-0.00038	-0.00057	-0.00056	-0.00056
C	-0.00064	-0.00158	-0.00039	-0.00105	-0.00144	-0.00055	-0.0002	-0.0002
D	-0.00084	-0.00125	-0.00083	-0.0009	0.00012	-0.00085	-0.0007	-0.0007
E	0.00005	-0.00084	-0.00027	-0.00083	-0.00054	-0.00096	-0.0005	-0.0005
F	0.00104	0.00128	0.00119	0.00047	0.00052	0.00084	0.00066	0.00066
G	-0.00034	-0.00194	-0.00136	-0.00072	-0.00081	-0.00016	0.00001	0.00001
H	0.00099	-0.00053	-0.00048	-0.00015	-0.0005	-0.00076	-0.00001	-0.00001
I	0.00022	-0.00035	-0.00029	-0.00022	-0.00016	-0.00032	0.00002	0.00002
K	-0.00241	-0.00144	-0.00151	-0.00176	-0.00036	-0.00108	-0.00085	-0.00085
L	-0.00182	-0.00108	-0.00127	-0.00124	-0.00132	-0.00081	-0.00039	-0.00039
M	-0.00135	-0.00109	-0.00151	-0.00102	-0.00072	-0.00104	-0.0005	-0.0005
N	-0.00174	-0.00137	-0.00097	-0.00121	-0.0011	-0.00052	-0.00067	-0.00067
P	0.00086	-0.00112	-0.00115	-0.00093	-0.00041	0.00012	-0.00028	-0.00028
Q	-0.00069	-0.00035	-0.00104	-0.00087	-0.00094	-0.00119	-0.0008	-0.0008
R	-0.00105	-0.00152	-0.001	-0.00124	-0.00053	-0.00059	-0.00045	-0.00045
S	-0.00104	-0.00093	-0.00098	-0.00059	-0.00087	-0.00052	-0.00025	-0.00025
T	-0.00054	-0.00046	-0.00114	-0.00097	-0.001	-0.00065	-0.00062	-0.00062
V	-0.00095	-0.00271	-0.00067	-0.00102	-0.00131	-0.00109	-0.0012	-0.0012
W	-0.00062	-0.0015	-0.00045	-0.00092	-0.00045	-0.00072	-0.00044	-0.00044
Y	-0.00075	0.00000	-0.00057	-0.00047	-0.00054	-0.0013	-0.00012	-0.00012

Interaction #7

V3 (13)	bin1	bin2	bin3	bin4
A	-0.00069	-0.00047	-0.00068	-0.00085
C	-0.00058	-0.00047	-0.00067	-0.00025
D	-0.00041	0.00008	-0.00009	-0.00046
E	-0.00049	-0.00086	0.00006	-0.00087
F	-0.00118	-0.00104	-0.00093	-0.00098
G	-0.00053	-0.00127	-0.00046	0.00009
H	-0.00086	-0.00123	-0.00037	-0.00052
I	0.00049	0.00062	0.00104	-0.00026
K	-0.00093	0.00002	0.00037	0.00001
L	0.00005	0.00043	0.00087	0.00038
M	-0.00028	-0.00053	-0.0005	-0.00059
N	-0.00081	-0.00074	-0.00048	-0.00036
P	-0.00131	0.00131	-0.00031	-0.00047
Q	-0.00135	-0.00078	-0.00093	0.00013
R	0.00033	-0.00044	-0.00046	-0.00053
S	-0.0004	0.00068	0.00043	-0.0003
T	-0.00004	0.00037	-0.00025	-0.00054
V	-0.00027	-0.00173	-0.00004	0.00043
W	-0.00052	-0.00013	-0.00015	-0.00013
Y	0.00008	-0.00029	0.00008	-0.00042

Interaction #8

V3 (13)	bin4	bin9
A	-0.00051	0.00018
C	-0.00049	-0.00003
D	-0.00029	-0.00022
E	-0.0002	-0.00018
F	-0.00151	-0.0005
G	0.00004	0.00015
H	-0.00043	-0.00051
I	-0.00042	-0.00021
K	0.00001	-0.00015
L	-0.00095	-0.00042
M	-0.00149	-0.00025
N	-0.00052	-0.00033
P	-0.001	-0.0003
Q	-0.00059	0.00011
R	0.00005	0.00023
S	-0.00059	-0.00001
T	0.00021	0.00063
V	-0.00138	0.00027
W	-0.00053	0.00020
Y	-0.00038	0.00020

Interaction #9

V3 (18)	bin3	bin4	bin5
A	0.00045	-0.0002	-0.00006
C	-0.00076	0.00025	0.00058
D	-0.00006	0.00011	0.00016
E	0.00071	0.00058	0.00049
F	0.00000	-0.00057	-0.00047
G	-0.00126	-0.00088	-0.00055
H	0.00018	0.00064	0.00057
I	0.00066	0.00083	0.00026
K	0.00111	0.00124	0.00053
L	-0.0009	-0.00061	-0.00056
M	-0.0008	-0.00038	-0.00011
N	-0.00005	0.00009	0.00033
P	-0.00084	0.00010	0.00004
Q	-0.0011	-0.00077	-0.00024
R	0.00099	0.00136	0.00079
S	0.00008	-0.00042	0.00044
T	0.00095	0.00084	0.00085
V	-0.00141	-0.00014	-0.00007
W	-0.00077	-0.00049	-0.00015
Y	-0.0009	-0.00078	-0.0002

Interaction #10

V3 (18)	bin1	bin2	bin3
A	0.00014	0.00059	0.00045
C	-0.00193	-0.00067	-0.00076
D	0.00065	-0.00022	-0.00006
E	0.00098	0.00070	0.00071
F	-0.00052	-0.00075	0.00000
G	-0.0017	-0.00141	-0.00126
H	0.00057	0.00062	0.00018
I	0.00081	-0.00009	0.00066
K	-0.00063	-0.00043	0.00111
L	-0.00089	-0.00072	-0.0009
M	-0.00092	-0.00109	-0.0008
N	0.00039	0.00004	-0.00005
P	-0.0006	-0.00037	-0.00084
Q	-0.00138	-0.00165	-0.0011
R	0.00125	0.00121	0.00099
S	0.00008	-0.00029	0.00008
T	0.00097	0.00061	0.00095
V	-0.00046	-0.00053	-0.00141
W	-0.0006	-0.00102	-0.00077
Y	-0.00047	-0.00102	-0.0009

Interaction #11

V3 (22)	bin1	bin3
A	-0.00015	-0.00044
C	-0.00014	0.00004
D	-0.00149	-0.00022
E	-0.00106	-0.0006
F	-0.00039	-0.00125
G	-0.00036	-0.0009
H	-0.0013	0.00000
I	-0.00006	-0.00067
K	0.00040	0.00082
L	-0.00055	0.00066
M	-0.00036	-0.0001
N	-0.00186	-0.00068
P	-0.00134	0.00064
Q	-0.0001	0.00020
R	-0.00008	-0.00043
S	-0.00041	-0.00009
T	-0.00064	-0.00002
V	-0.00082	-0.0002
W	-0.00001	-0.00036
Y	0.00065	-0.00006
